# Homozygous *FOXE3* mutations cause non-syndromic, bilateral, total sclerocornea, aphakia, microphthalmia and optic disc coloboma

**Published:** 2010-06-23

**Authors:** Manir Ali, Beatriz Buentello-Volante, Martin McKibbin, J. Alberto Rocha-Medina, Narcis Fernandez-Fuentes, Wilson Koga-Nakamura, Aruna Ashiq, Kamron Khan, Adam P. Booth, Grange Williams, Yasmin Raashid, Hussain Jafri, Aine Rice, Chris F. Inglehearn, Juan Carlos Zenteno

**Affiliations:** 1 Section of Ophthalmology and Neuroscience, Leeds Institute of Molecular Medicine, St. James's University Hospital, Leeds, UK; 2 Research Unit, Institute of Ophthalmology “Conde de Valenciana,” Mexico City, Mexico; 3 Eye Department, Chancellor Wing, St James's University Hospital, Leeds, UK; 4 Section of Experimental Therapeutics, Leeds Institute of Molecular Medicine, St. James's University Hospital, Leeds, UK; 5 Echography Service, Institute of Ophthalmology “Conde de Valenciana,” Mexico City, Mexico; 6 Department of Ophthalmology, Manchester Royal Eye Hospital, Manchester, UK; 7 Peninsula Medical School, Plymouth, UK; 8 Department of Obstetrics and Gynaecology, King Edward Medical University, Lahore, Pakistan; 9 Gene Tech Lab 146/1, Shadman Jail Road, Lahore, Pakistan; 10 Department of Biochemistry, Faculty of Medicine, National Autonomous University of Mexico, Mexico City, Mexico

## Abstract

**Purpose:**

To investigate the genetic basis of recessively-inherited congenital, non syndromic, bilateral, total sclerocornea in two consanguineous pedigrees, one from the Punjab province of Pakistan and the other from the Tlaxcala province of Mexico.

**Methods:**

Ophthalmic examinations were conducted on each family member to confirm their diagnosis and magnetic resonance imaging (MRI) or ultrasonography of the eyes was performed on some family members. Genomic DNA was analyzed by homozygosity mapping using the Affymetrix 6.0 SNP array and linkage was confirmed with polymorphic microsatellite markers. Candidate genes were sequenced.

**Results:**

A diagnosis of autosomal recessive sclerocornea was established for 7 members of the Pakistani and 8 members of the Mexican pedigrees. In the Pakistani family we established linkage to a region on chromosome 1p that contained Forkhead Box E3 (*FOXE3*), a strong candidate gene since *FOXE3* mutations had previously been associated with various anterior segment abnormalities. Sequencing *FOXE3* identified the previously reported nonsense mutation, c.720C>A, p.C240X, in the Pakistani pedigree and a novel missense mutation which disrupts an evolutionarily conserved residue in the forkhead domain, c.292T>C, p.Y98H, in the Mexican pedigree. Individuals with heterozygous mutations had no ocular abnormalities. MRI or ultrasonography confirmed that the patients with sclerocornea were also aphakic, had microphthalmia and some had optic disc coloboma.

**Conclusions:**

This is the fourth report detailing homozygous *FOXE3* mutations causing anterior segment abnormalities in human patients. Previous papers have emphasized aphakia and microphthalmia as the primary phenotype, but we find that the initial diagnosis – and perhaps the only one possible in a rural setting – is one of non-syndromic, bilateral, total sclerocornea. Dominantly inherited anterior segment defects have also been noted in association with heterozygous *FOXE3* mutations. However the absence of any abnormalities in the *FOXE3* heterozygotes described suggests that genetic background and environmental factors plays a role in the penetrance of the mutant allele.

## Introduction

Sclerocornea is a nonprogressive, non inflammatory developmental anomaly in which the normal scleral tissue extends beyond the limbus into the peripheral cornea, causing opacification and vascularization (scleralization) [1]. It is usually bilateral, although it can be asymmetric, and can vary in severity from total opacification of the cornea, which limits the visualization of intraocular structures, to mild peripheral corneal vascularization. It is thought to result from a disordered migration of neural crest cells between the corneal epithelium and endothelium during fetal development [2,3]. Sclerocornea is usually seen in sporadic cases but familial clustering is also well documented, with recessive inheritance leading to a more severe phenotype than the dominant form [4,5]. The condition can occur alone, in association with other ocular symptoms or with systemic features as part of a syndrome.

The genes implicated in sclerocornea include *FOXE3* (Forkhead Box E3; OMIM 601094) on chromosome 1p. Mutations in *FOXE3* cause recessive sclerocornea in association with microphthalmia, bilateral aphakia, absence of the iris and retinal dysplasia (OMIM 610256) [5-7]. Other mutations in the same gene cause dominant disease with various anterior segment abnormalities [4,6,8]. In addition mutations in the *Rax* gene (OMIM 601881) on chromosome 18q caused sclerocornea, anophthalmia and autism (OMIM 611038) in a 12-year-old boy born to non-consanguineous parents [9]. The defective genes remain to be identified for a locus on Xp22.31, which is associated with microphthalmia-dermal aplasia-sclerocornea syndrome (MIDAS; OMIM 309801) [10], for sclerocornea associated with 22q11.2 deletion syndrome [11] and for an interstitial deletion on chromosome 6p in a dysmorphic infant [12]. The purpose of this study was to look at two consanguineous pedigrees with multiple affected members that appear to have a homogeneous phenotype of congenital, non-syndromic, bilateral, total sclerocornea, to determine the underlying genetic abnormality.

## Methods

### Patients

**Figure 1:**
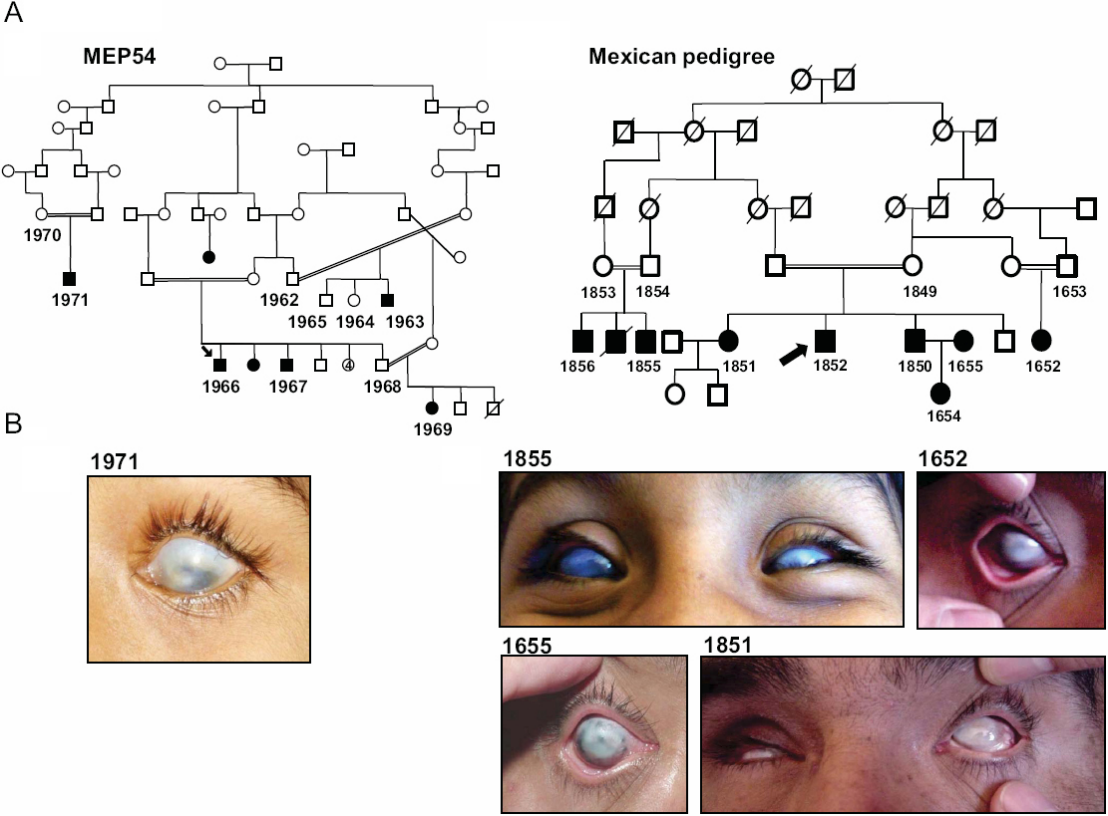
Clinical description of the families. **A**: The pedigree structures are shown for MEP54 and the Mexican pedigree. Affected individuals are depicted with filled-in symbols. The numbers highlight the family members from whom blood was taken for DNA extraction. **B**: Anterior segment photos were taken using a Nixon Camera for affected member 1971 (aged 8 years) from the Pakistani and 1855 (aged 12 years), 1652 (aged 40 years), 1655 (aged 38 years) and 1851 (aged 48 years) from the Mexican pedigrees. Note total sclerocornea. Acuity was hand movements only.

Participants in this study gave informed consent in accordance with the tenets of the Declaration of Helsinki, using a protocol approved by the Leeds East Ethics committee and the “Conde de Valenciana” Institute ethics committee. The pedigrees ascertained were from Multan in the Punjab province of Pakistan and the Tlaxcala province of Mexico. A detailed family history revealed that within each pedigree the families were connected by multiple consanguineous loops (see Figure 1A). Corneal opacities were present from birth or very early in life in the affected subjects and none of the patients had previous ophthalmic surgery. Ophthalmic examination revealed complete corneal opacification and absence of the corneal limbus. Multi-plane, sequential magnetic resonance imaging of the brain and orbit and conventional eye ultrasonography was performed in some patients.

### Homozygosity mapping

DNA aliquots from five affected members of the Pakistani family were mixed in equal amounts to form a single sample, which was genotyped on a single Affymetrix 6.0 SNP array (AROS Applied Biotechnologies, Aarhus, Denmark). Regions of homozygosity were highlighted by conditional formatting in excel. Linkage was confirmed with fluorescently-labeled polymorphic microsatellite markers on a 3130xl Genetic Analyzer (Applied Biosystems, Warrington, UK) using GeneMapper version 4.0 (Applied Biosystems).

### DNA sequencing

Specific primer pairs for the amplification of the coding regions as well as the exon-intron boundaries, of the *FOXE3* gene are presented in Table 1. PCR products were digested with ExoSAP-IT (GE Healthcare, Chalfont St. Giles, UK) and sequenced using the BigDye Terminator version 3.1 Cycle Sequencing Kit. Products were resolved on a 3130xl Genetic Analyzer according to the manufacturer’s instructions (Applied Biosystems).

**Table 1:** Oligonucleotide primer pairs for the amplification of the *FOXE3* gene.

**Amplicon**	**Forward primer**	**Reverse primer**	**Size of PCR product (bp)**	**Temp. (°C)**
1	TTGGGAATGATCCAAAGGAG	GGCAGGGAAGCCAGAGAA	400	56
2	GGGGCCGTGTCCATATAAAG	CCGCTGCCGTTGTCGAAC	554	58
3	AACGACTGCTTCGTCAAGGT	GCGCAGGCTCACAGGTGAG	593	58
4	TGGGGAGGCCTACCTGAG	ACTCACTGGAGGCGAGTCA	392	56
5	ACAGAAGCGTCCCCTTTGAC	AGGCAGCCAGGTGTGTCTAC	398	58
6	TCCTGGGTTCATGACTTACTTG	CCATGTGGCAACCCAAGAT	397	56

### Bioinformatics

Conservation of protein sequence across mammalian and non-mammalian vertebrates was investigated at the University of California Santa Cruz (UCSC) Genome Browser using the Vertebrate Multiz Alignment and PhastCons Conservation package.

### Molecular modeling

The sequences of the forkhead domain of human FOXE3 wild type (Uniprot accession number Q13461) and Y98H mutant were modeled using our previously described method [13]. Two templates were used to model the target sequences: the DNA-binding domain of the human transcription factor FREAC-11 [14] and DNA-binding domain of rat FoxE3 [15]. The average sequence identity between target sequences and templates was >75%, assuring high-fidelity structural modeling [16]. The quality of the models was assessed using ProSA-II [17] and PROCHECK [18].

## Results

We studied two consanguineous pedigrees, one from the Punjab province of Pakistan and the other from the Tlaxcala province of Mexico, with multiple blind members each of whom had a poorly defined cornea with no anatomic limbal boundary between the cornea and sclera. Patients had no neurologic or systemic abnormalities so the diagnosis was established as non-syndromic, bilateral, total sclerocornea (Figure 1). The absence of any anterior segment abnormalities in the parents of the affected individuals, together with the presence of multiple affected siblings within a family, suggested recessive inheritance.

**Figure 2:**
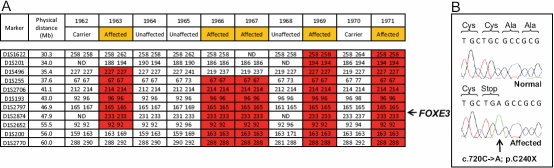
Molecular analysis of the Pakistani pedigree. **A:** Confirmatory microsatellite genotyping highlighted a homozygous region on chromosome 1q between the markers D1S496 and D1S200 as being linked with the disease phenotype. The physical distance for each marker is represented based on the human February 2009 assembly (hg19) of the UCSC Genome Browser. The *FOXE3* gene is marked within the refined interval at 47.9Mb. **B**: The sequencing chromatogram shows the c.720C>A mutation in the *FOXE3* gene in an affected member of the Pakistani pedigree.

Whole genome SNP genotyping in the Pakistani pedigree revealed a 20.6 Mb region on chromosome 1p between the markers D1S496 and D1S200 which was linked with the disease phenotype (Figure 2A). Within this interval was the *FOXE3* gene, which encodes a transcription factor, that is expressed in the formation of the lens placode during embryonic development [19,20] and is confined to the anterior lens epithelium in adults [6,19]. Furthermore, *FOXE3* mutations have been found in patients with sclerocornea, microphthalmia, and bilateral aphakia [5-7]. Upon sequencing *FOXE3* in an affected member of the Pakistani family, we identified the previously reported homozygous C→A mutation that replaced the normal cysteine residue with a stop codon at the 240th amino acid in the protein (c.720C>A, p.C240X; Figure 2B).

**Figure 3:**
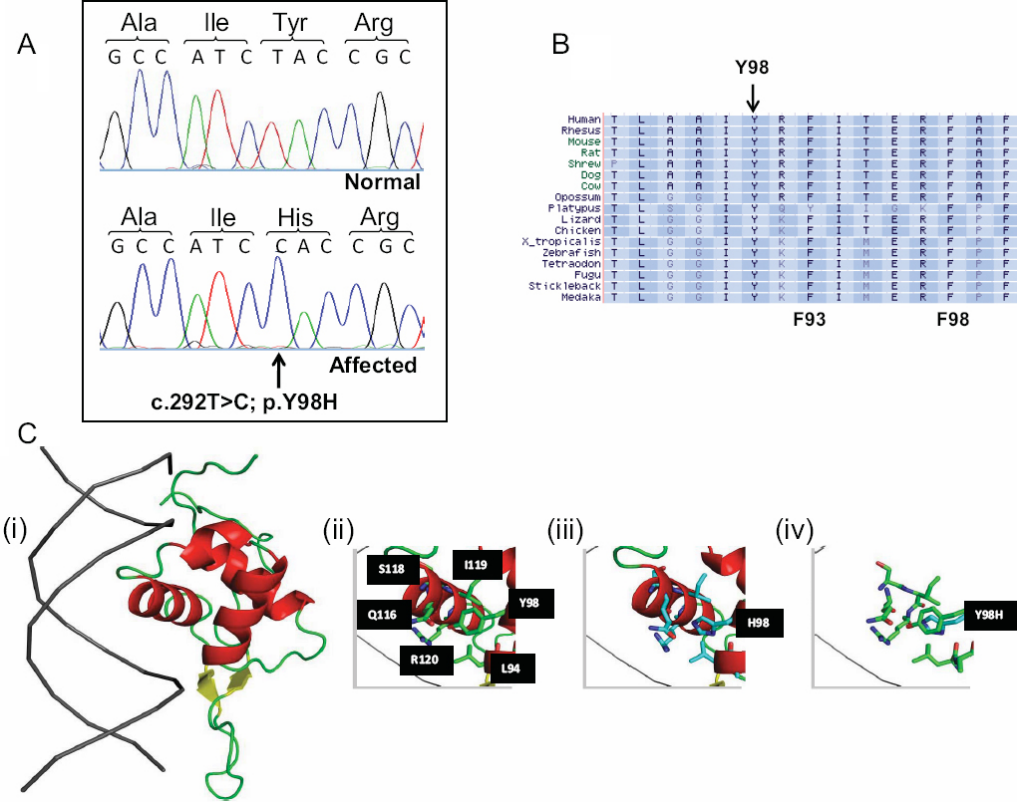
Molecular analysis of the Mexican pedigree. **A**: Sequencing chromatogram showing the c.292T>C mutation in the *FOXE3* gene in an affected member of the Mexican pedigree. **B**: Protein sequence conservation. Diagram showing part of the amino acid sequence of the FOXE3 protein within the forkhead domain. Note the evolutionary conserved tyrosine (Y) residue in the normal sequence that is mutated to a histidine in the patients with sclerocornea. The F93 and F98 residues that are mutated to give rise to the *dysgenetic lens* mouse mutant are also depicted. **C**: Structural model of the forkhead domain of human FOXE3 wildtype and p.Y98H. (i) Ribbon representation of the DNA – fork head domain complex. DNA is depicted in gray and forkhead domain in red, yellow or green depending if helix, beta strand or loop regions. Structural microenvironment of Y98 (ii) and H98 (iii), residues within 6 Angstrom of Y98, or H98, is shown in stick representation and labeled in black boxes. (iv) Structural overlay of wild type (Y98) and mutant (H98). Figures were generated using PyMOL.

As the Mexican patients presented with a similar phenotype, the *FOXE3* gene was sequenced and a homozygous T→C mutation was identified, resulting in a non-conservative substitution of the 98th residue in FOXE3 from a tyrosine to a histidine (c.292T>C, p.Y98H; Figure 3A). This mutation, which disrupts an evolutionarily conserved residue (Figure 3B), was shown by direct sequencing of genomic DNA to segregate with the disease phenotype in the pedigree (data not shown) and was also absent from 250 control chromosomes. This mutation is only the second human missense mutation within the *FOXE3* forkhead domain that has been reported in a recessive phenotype. At the molecular level, the mutation replaces a neutral, polar side group in the tyrosine residue of the normal protein with a positively charged imidazole side chain of histidine. This change in physical and chemical properties of the amino acid residue alters the local structural microenvironment around the alpha helix that usually interacts with the major groove of DNA, and is likely to abrogate the DNA binding capacity of the protein (Figure 3C).

**Figure 4:**
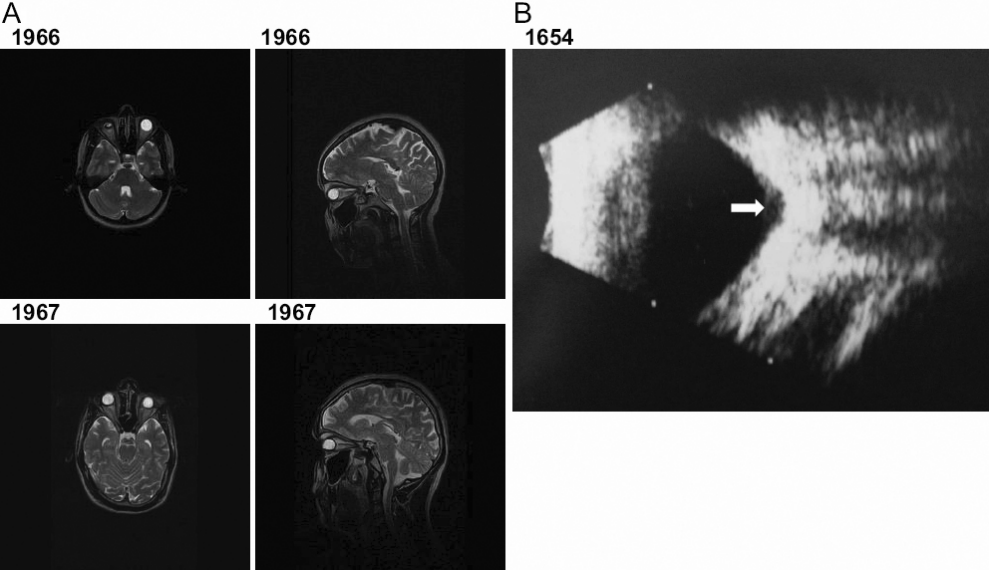
Retrospective analysis of the patients with sclerocornea for further ocular abnormalities. **A**: Axial and sagittal T2-weighted MRI scans of the head and orbits of two affected members 1966 and 1967 (aged 25 and 22 years old) from the MEP54 pedigree. Both patients demonstrate aphakia as depicted by the absence of a dark lens in the anterior part of the eye. In the absence of surgery, the left eye of patient 1966 seems to be phthisical. The axial lengths for 1966 are 10 and 17 mm and 1967 are 19 and 16 mm for the right and left eyes, respectively confirming that there is also microphthalmia. The sagittal section shows that there are no obvious structural abnormalities of the brain. **B**: Left eye ultrasound scan of patient 1654 from the Mexican pedigree showing an optic disc coloboma (white arrow).

Since previous reports with homozygous *FOXE3* mutations described sclerocornea in association with aphakia and microphthalmia [5-7], magnetic resonance imaging (MRI) was used to examine the eyes in 2 affected individuals from the Pakistani family and ultrasound was used to examine the eyes in 5 affected individuals from the Mexican pedigree (Figure 4). MRI confirmed that affected individuals in these families are indeed aphakic and have shortened axial lengths (Figure 4A). Ultrasonography also demonstrated bilateral optic disc coloboma in 3 out of 5 Mexican affected subjects (Figure 4B). Based on clinical findings and family history, the disease segregating in these families was therefore classified as non-syndromic, bilateral, total sclerocornea, aphakia, microphthalmia and optic disc coloboma.

## Discussion

In this paper, we report two consanguineous pedigrees, one from Pakistan and the other from Mexico, that presented with congenital, non-syndromic, bilateral, total sclerocornea. Molecular investigations identified a previously documented mutation in *FOXE3*, c.720C>A, p.C240X, in the Pakistani pedigree and a novel *FOXE3* missense mutation, c.292T>C, p.Y98H, in the Mexican pedigree. Further clinical investigations confirmed aphakia, microphthalmia, and optic disc coloboma in affected members. Previous papers have emphasized aphakia and microphthalmia as the primary phenotype, but we find that the initial diagnosis – and perhaps the only one possible in a rural setting – is one of non-syndromic, bilateral, total sclerocornea. This is not surprising since *FOXE3* expression coincides with the formation of the lens placode during embryonic development [19,20], and mutations in genes that lead to lenticular abnormalities are likely to cause anterior segment disease and retinal defects.

This is the fourth publication reporting homozygous *FOXE3* mutations in patients with these ocular symptoms, bringing the total to nine families with six different mutations. The original report describing the c.292T>C, p.C240X mutation did not mention the ethnicity of the family involved [5], however a recent report also identified this mutation in patients from Bangladesh and Kuwait [7]. It is possible that this may represent a founder mutation with all the families related by a common distant ancestor. The second report of homozygous *FOXE3* mutations in human patients identified c.244A>G, p.M82V and c.21_24del, p.M71IfsX216 in two consanguineous pedigrees of Pakistani origin [6]. The third, and most recent report, also identified the c.244A>G, p.M82V mutation as a compound heterozygote with c.705delC, p.E236SfsX71 in a Caucasian patient and another homozygous mutation c.557delT, p.F186SfsX38 in a patient from United Arab Emirates [7].

Heterozygous mutations in *FOXE3* have also been implicated in dominant disease and may give rise to various anterior segment abnormalities [4,6,8]. These include a c.942dupG, p.L315AfsX117 mutation found in a mother and daughter with congenital cataract and posterior embryotoxon [4]; a c.269G>T, p.R90L mutation in a patient with Peter’s anomaly [8]; and c.885T>C, p.X320RextX72 in a family with Peter’s anomaly, early onset cataract and coloboma as well as a c.146G>C, p.G49A mutation in a family with microphthalmia, cerulean type cataracts and chorioretinal coloboma [6]. However, our study confirms previous observations [5-7] that highlight the absence of any ocular abnormalities in *FOXE3* heterozygotes from families with recessive disease, suggesting either that specific *FOXE3* mutations cause phenotypes with different inheritance modes or that genetic background as well as environmental factors may play a role in the penetrance of the mutant alleles.

It is interesting to note that *FoxE3* knock out mice [21] and the dysgenetic lens (*dyl*) mouse which has two homozygous missense mutations, p.F93L and p.F98S, in the forkhead domain [19,20], both form a small lens that fails to detach from the surface ectoderm during ocular development and so remains partly attached to the cornea. This phenotype is milder than the one described in humans with homozygous *FOXE3* mutations, who are aphakic. While heterozygote knockout mice are normal during embryonic development, some mutation carriers later develop keratolenticular adhesions as adults [21]. *Dyl* heterozygotes display corneal and lenticular abnormalities with incomplete penetrance, similar to Peter’s anomaly in humans [8]. These observations provide supportive evidence that the pathogenic effects of FoxE3 haploinsufficiency could be dependant on other modifiers that remain to be identified.

To conclude, we report that patients presenting with non-syndromic, bilateral, total sclerocornea ought to be screened for *FOXE3* mutations in the first instance. Our findings also highlight the absence of any obvious ocular abnormalities in the *FOXE3* heterozygotes, when examined in the rural setting, contrasting with previous reports where anterior segment defects have been associated with heterozygous *FOXE3* mutations. These observations suggest that genetic background and environmental factors may play a role in the penetrance of the mutant allele.
